# The Stem Cell Phenotype of Aggressive Breast Cancer Cells

**DOI:** 10.3390/cancers11030340

**Published:** 2019-03-08

**Authors:** Naira V. Margaryan, Hannah Hazard-Jenkins, Mohamad A. Salkeni, Matthew B. Smolkin, James A. Coad, Sijin Wen, Elisabeth A. Seftor, Richard E. B. Seftor, Mary J. C. Hendrix

**Affiliations:** 1 Department of Biochemistry, West Virginia University, Morgantown, WV 26506, USA; naira.margaryan@hsc.wvu.edu (N.V.M.); elisabeth.seftor@hsc.wvu.edu (E.A.S.); richard.seftor@hsc.wvu.edu (R.E.B.S.); 2 West Virginia University Cancer Institute, West Virginia University, Morgantown, WV 26506, USA; hhazard@hsc.wvu.edu (H.H.-J.); mosalkeni@hsc.wvu.edu (M.A.S.); mbsmolkin@hsc.wvu.edu (M.B.S.); jcoad@hsc.wvu.edu (J.A.C.); siwen@hsc.wvu.edu (S.W.); 3 Department of Surgery, West Virginia University, Morgantown, WV 26506, USA; 4 Department of Internal Medicine, West Virginia University, Morgantown, WV 26506, USA; 5 Department of Pathology, Anatomy and Laboratory Medicine, West Virginia University School of Medicine, Morgantown, WV 26506, USA; 6 Department of Biostatistics, School of Public Health, West Virginia University, Morgantown, WV 26506, USA; 7 Department of Biology, Shepherd University, Shepherdstown, WV 25443, USA

**Keywords:** Nodal, breast cancer, ABCA1, cancer stem cells, doxorubicin/cyclophosphamide/taxanes (paclitaxel or docetaxel) (ACT), docetaxel/carboplatin/trastuzumab/pertuzumab (TCHP), docetaxel/cyclophosphamide (TC)

## Abstract

Aggressive cancer cells are characterized by their capacity to proliferate indefinitely and to propagate a heterogeneous tumor comprised of subpopulations with varying degrees of metastatic propensity and drug resistance properties. Particularly daunting is the challenge we face in the field of oncology of effectively targeting heterogeneous tumor cells expressing a variety of markers, especially those associated with a stem cell phenotype. This dilemma is especially relevant in breast cancer, where therapy is based on traditional classification schemes, including histological criteria, differentiation status, and classical receptor markers. However, not all patients respond in a similar manner to standard-of-care therapy, thereby necessitating the need to identify and evaluate novel biomarkers associated with the difficult-to-target stem cell phenotype and drug resistance. Findings related to the convergence of embryonic and tumorigenic signaling pathways have identified the embryonic morphogen Nodal as a promising new oncofetal target that is reactivated only in aggressive cancers, but not in normal tissues. The work presented in this paper confirms previous studies demonstrating the importance of Nodal as a cancer stem cell molecule associated with aggressive breast cancer, and advances the field by providing new findings showing that Nodal is not targeted by standard-of-care therapy in breast cancer patients. Most noteworthy is the linkage found between Nodal expression and the drug resistance marker ATP-binding cassette member 1 (ABCA1), which may provide new insights into developing combinatorial approaches to overcome drug resistance and disease recurrence.

## 1. Introduction

Decades’ worth of research has sought to define the various stages of breast cancer progression with the overall goal of improving the prediction of disease outcome. From a traditional perspective, the diagnosis of breast cancer has relied heavily on histological criteria [1]. In addition, the Nottingham grading system has specified key architectural features that further define invasive breast cancer and are critical to classifying the differentiation status, with a poorly differentiated phenotype being the hallmark of more aggressive disease [2]. Fortunately, advances in molecular medicine have revealed critical markers, such as estrogen receptor (ER), progesterone receptor (PR), and human epidermal growth factor receptor 2 (Her2), and these have been especially helpful in further classifying certain types of breast cancers into distinctive functional groups [3], in addition to informing selection of patients for specific targeted treatment options [4,5].

Poorly differentiated, aggressive breast cancer cells have been shown to possess stem cell properties, proliferate indefinitely, and propagate a tumor comprised of heterogeneous cell subpopulations with varying degrees of drug resistance and metastatic properties [6]. One of the most daunting challenges we face in the oncological sciences is developing the most effective targeting of these heterogeneous tumors containing breast cancer cells expressing various markers, especially those associated with stem cells, for which targeted therapies are currently under development. Particularly noteworthy is the phenotype associated with aggressive triple-negative breast cancer (TNBC), which exhibits little-to-no expression of classical markers, and patients are at significantly higher risk of relapsing with metastatic disease following treatment with standard-of-care therapies. Thus, there is a critical need to identify novel targetable molecules that can enhance current therapies by mitigating the stem cell phenotype of aggressive breast cancer and concurrently reversing drug resistance.

Tumor cell plasticity underlies the stem cell phenotype. Indeed, our basic understanding of the functional properties associated with this phenotype has been significantly advanced by the molecular analyses of aggressive breast cancer cells compared with nonaggressive breast cancer [7,8]. Particularly noteworthy for the aggressive phenotype is the co-expression of multiple cell type-specific markers normally associated with endothelial cells, mesenchymal cells, and stem cells, concomitant with the downregulation of E-cadherin, ER, and PR [6]. Further functional analyses have uncovered selective advantages associated with this plastic phenotype pertinent to tumorigenesis, metastasis, and drug resistance, including epithelial-to-mesenchymal transition (EMT), vasculogenic mimicry (VM), and unregulated growth potential via the reactivation of the Nodal signaling pathway [9,10,11].

Due to Nodal’s quintessential role as a highly influential morphogen during critical phases of embryogenesis, together with the concept that tumorigenesis recapitulates many developmental events, our laboratory has focused attention on the implications of Nodal’s re-emergence in aggressive forms of cancer. From developmental biology studies, we know that Nodal is a member of the transforming growth factor-β (TGFβ) superfamily [12], and orchestrates the coordination of body axis formation, left-right patterning, the maintenance of human embryonic stem cell (hESC) pluripotency, and activation of EMT [12]. Nodal signals via binding to Cripto-1/ALK4/7/ActRIIB receptor complex, leading to the phosphorylation of Smad2/Smad3 followed by association with Smad4, and subsequent translocation to the nucleus [13]. Nodal expression in humans is largely restricted to embryonic tissues and is generally absent in normal adult tissues, rendering it a promising target specific to cancer. Based on Nodal’s critical role in sustaining the pluripotent phenotype of hESCs, we have hypothesized that it serves as a master plasticity gene in cancer, and have validated its expression as a cancer stem cell (CSC) signaling molecule with significant promise as a new target in aggressive forms of cancer [6,14,15]. In this regard, our laboratory and others have shown that Nodal expression underlies unregulated growth, tumorigenicity, and metastasis [11,12,14,16,17], and in terms of Nodal-positive, metastatic breast cancer, we and others have observed the concurrent expression of several stem cell markers, including CD44, ALDH1, Notch, Oct4, CD133, Sox2, and Nanog [18,19]. Furthermore, there is a correlation between a high level of Nodal expression and the expression of CD44 and lower overall survival of breast cancer patients [19]. The current paper focuses on the significance of Nodal expression in breast cancer patients who have undergone surgical intervention and standard-of-care therapy, and provides new insights into the association of this CSC molecule and drug resistance.

## 2. Results and Discussion

A previous, noteworthy breast cancer study from our laboratory focused on Nodal localization in 431 therapeutically naïve patients diagnosed with benign or malignant disease and revealed a potential role for Nodal as a new prognostic biomarker for disease progression when compared with currently used reference markers [16]. Specifically, the intensity of Nodal immunohistochemistry (IHC) staining was significantly stronger in undifferentiated, advanced stage invasive breast cancer compared with early stage breast disease. Treatment of human breast cancer cells in vitro with a Nodal blocking antibody results in reduced proliferation and diminished colony-forming ability. Experimental knockdown of Nodal in in vivo models of TNBC also results in significantly reduced levels of tumorigenesis [19]. These findings prompted the translationally relevant question whether Nodal is targeted by standard-of-care therapy in breast cancer patients. Based on the observations derived from our previous studies in melanoma patients and related animal models—where treatment with conventional dacarbazine or BRAF inhibitors did not diminish Nodal expression [14,17]—we postulated that Nodal would remain before and after standard-of-care therapy in breast cancer patients.

### 2.1. Nodal is Associated with Disease Progression

**Figure 1:**
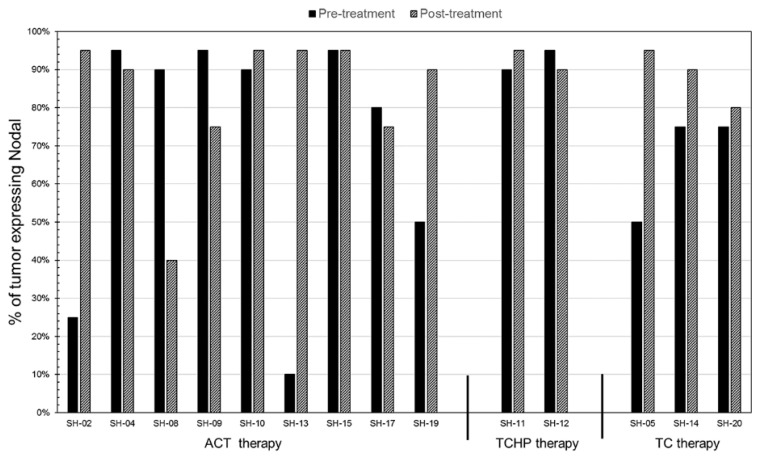
Clinically derived tissue sections from breast cancer patients, both pre- and post-treatment with current standard-of-care therapy (ACT, TCHP, and TC), were examined for the presence of Nodal protein by immunohistochemistry. The results are presented as a graph representing the percent of the tumor(s) that expresses Nodal categorized by the therapeutic treatment. Percent of tumor expressing Nodal is relative to Nodal measured in the entire tumor where 100% represents every tumor cell expressing Nodal.

**Figure 2:**
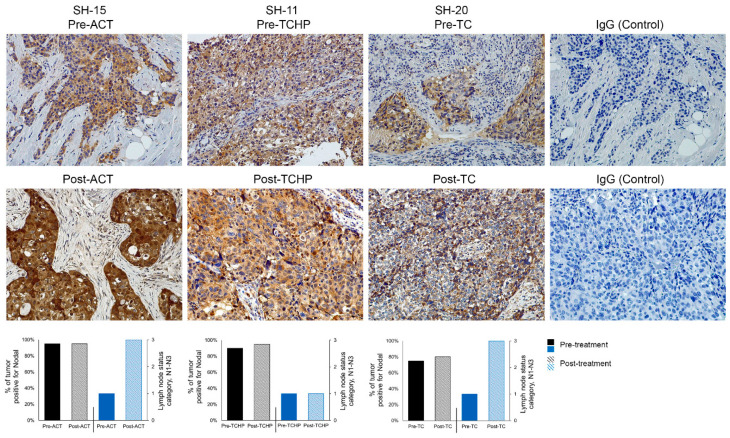
The presence of the Nodal protein (brown color) in breast cancer patient tumor sections, pre- and post-current-standard-of-care-treatments (ACT, TCHP, and TC), was examined by immunohistochemical staining with IgG used as a control for non-specific staining (20× original magnification). Bar graphs below the immunohistochemistry (IHC) data depict the percent of tumor positive for Nodal pre- and post-therapy (relative to where 100% represents every tumor cell expressing Nodal) correlated with the lymph node status categories N0–N3 (see Table 1 and Table 2 legends).

Tissue sections from 14 patients determined to have ductal carcinoma of the breast were studied before and after neoadjuvant therapy using IHC to evaluate Nodal expression, using a previously established scoring index [16]. Patient clinical characteristics are presented in Table 1 and Table 2, including the patients’ age; histological description; grade; clinical and pathological stage; original and post-therapy tumor size; status of ER, PR, Her2/neu, and Ki67; lymph node involvement before and after therapy; neoadjuvant standard-of-care therapy; surgical intervention; distant recurrence; years since diagnosis; and IHC scores for Nodal pre- and post-treatment. The Nodal IHC analysis of these patients’ tumors, pre- and post-treatment with current standard-of-care therapy (doxorubicin/cyclophosphamide/taxanes (ACT), docetaxel/carboplatin/trastuzumab/pertuzumab (TCHP) and docetaxel/cyclophosphamide (TC)), is presented as a graph in Figure 1 showing the percent of the tumor that expresses Nodal relevant to the therapeutic treatment. In all cases, Nodal is expressed before and after treatment and, in the majority of cases, Nodal expression is enhanced post-treatment. Although a power assessment is not possible to achieve with this small sample number, the data shown support the hypothesis that Nodal is not abrogated by standard-of-care therapy. Nodal IHC staining is presented in Figure 2 in three patients’ tumors, where Nodal expression appears enhanced following treatment with ACT, TCHP, or TC. Further analysis suggests that there might be an increase in lymph node involvement with enhanced Nodal expression and supports the need for further studies to identify if there is a correlation between Nodal expression and disease progression in a larger patient cohort.

**Figure 3:**
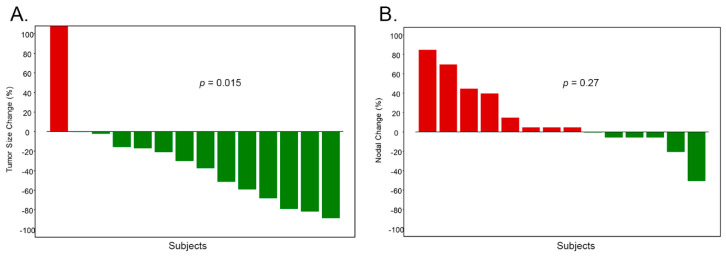
The standard-of-care treatments used in the study (ACT, TCHP, and TC) decreased the size of the tumors but did not change the percent of Nodal in the tumors. (**A**) There is a statistically significant decrease in the tumor size in response to the different standard-of-care treatments (*p* = 0.015, Wilcoxon signed-rank test); while (**B**) there is a statistically insignificant change in the percent of Nodal in the tumors after treatment (*p* = 0.27, Wilcoxon signed-rank test).

While there is a statistically significant decrease in the tumor size in response to the different standard-of-care treatments (ACT, TCHP and TC; Figure 3A; *p* = 0.015), there is a statistically insignificant change in the percent of Nodal in the tumors after the different treatments (Figure 3B; *p* = 0.27). These observations suggest that while these standard-of-care treatments do kill some cells in the tumor population, there appears to be a subpopulation of tumor cells that is drug resistant and expresses Nodal.

### 2.2. Nodal is Associated with Drug Resistance

**Figure 4:**
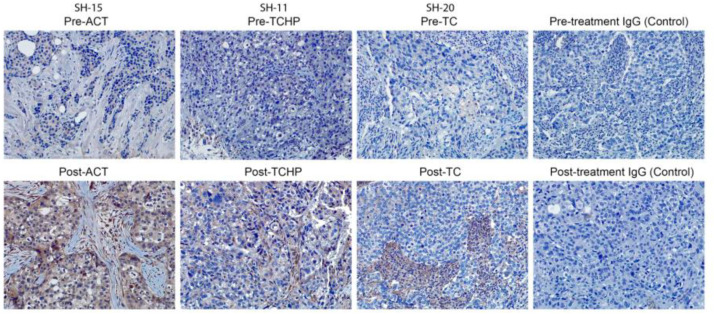
The presence of the ABCA1 protein (brown color) in breast cancer patient tumor sections, pre- and post-current-standard-of-care-treatments (ACT, TCHP, and TC) was examined by immunohistochemical staining with IgG used as a control for non-specific staining (20× original magnification).

In previous melanoma studies, we observed an association between Nodal expression and the drug resistance marker ABCA1 [20]. Most noteworthy, a direct correlation was demonstrated when Nodal expression was downregulated, resulting in the complete mitigation of ABCA1. These data provided the first direct evidence linking the CSC signaling molecule Nodal and drug resistance, which provided new insights into the possible mechanisms underlying Nodal’s significance in aggressive cancer. We extended these observations to the current breast cancer study where, using tissue sections from the same patients’ tumors shown in Figure 2 for Nodal staining and lymph node involvement, we performed IHC localization for ABCA1, as presented in Figure 4. The IHC staining pattern for ABCA1 is particularly noteworthy in the post-treatment samples for ACT, TCHP, and TC therapy, and may provide new clues linking patient responsiveness relevant to these therapies. Furthermore, sections from these same samples were also dual stained for both Nodal and ABCA1 and cells identified which stain for both Nodal and ABCA1 (Supplemental Figure S1).

The ABCA1 protein is an ATP-binding cassette transporter which functions as a cholesterol efflux pump in the cellular lipid removal pathway and acts as the primary gatekeeper for eliminating tissue cholesterol [21]. Interestingly, ABCA1 has been shown to be upregulated in drug resistance to curcumin in melanoma [22], doxorubicin resistance in breast cancer and hepatocellular carcinoma [23,24], paclitaxel and carboplatin resistance in serous epithelial ovarian cancer [25], and cisplatin resistance in non-small-cell lung and epidermoid carcinomas [26]. Particularly noteworthy is the Kaplan–Meier plot for ABCA1 low and high expression in breast cancer patient tumors—documented in the National Center for Biotechnology Information (NCBI), USA National Library of Medicine database, which shows a significantly better survival probability for patients expressing low levels of ABCA1 in their breast cancer compared with those who have a high expression [27].

Collectively, these observations—showing a connection between Nodal’s presence in aggressive breast cancer, before and after standard-of-care therapy, with a possible association with the ABCA1 drug resistance marker—provide preliminary evidence for pursuing in more depth. Since Nodal acts as a CSC signaling molecule and is only expressed by subpopulations within a heterogeneous tumor, our body of experimental data supports a future therapeutic approach using standard-of-care therapy in a combinatorial manner with anti-Nodal therapy. We have tested this approach in three experimental TNBC models expressing high levels of Nodal, where treatment with doxorubicin did not effectively diminish the Nodal target [11]. However, sequential treatment of the TNBC models with doxorubicin, followed by anti-Nodal antibody regimen, resulted in significant decreases in cellular growth and viability. This study further revealed that anti-Nodal antibody treatment, following doxorubicin, affects the cellular stress (p38) and repair (ChK1) pathways. These findings support a unique approach in inhibiting Nodal, thereby disrupting the cancer cell’s ability to repair their compromised DNA following front-line therapy. Additional studies focused on Nodal’s role in drug resistance are warranted to better predict patients’ responsiveness to standard-of-care therapy.

## 3. Materials and Methods

### 3.1. Breast Cancer Patient Samples

Archival formalin-fixed and paraffin-embedded breast tissue sections from 14 patients diagnosed with ductal breast cancer (with varying ER, PR status, and Her2 expression) were matched relevant to pre- and post-treatment with standard-of-care therapy (ACT, TCHP, or TC), and obtained from the Betty Puskar Breast Care Center, Morgantown, West Virginia. Patient tumor sections were de-identified and labeled with numerical codes in accordance with the approved West Virginia University expedited IRB protocol (#1705572966R00) for using de-identified patient samples for research.

### 3.2. Immunohistochemistry

Four-micron-thick, formalin-fixed, paraffin-embedded tissue sections were prepared and immunohistochemistry was carried out on a DAKO AutostainerPlus (AgilentTechnologies Inc., Santa Clara, CA, USA), as previously described [14]. Briefly, following antigen retrieval and blocking steps, sections were incubated with a mouse anti-human Nodal antibody (Abcam, ab55676, 1:300; Cambridge, MA, USA) or rabbit ABCA1 antibody (Novus Biologicals, NB400-105, 1:200; Centennial, CO, USA) for 60 min, followed by biotinylated anti-mouse or anti-rabbit secondary antibody, respectively (GM601 and GM608, Biocare Medical, LLC, Concord, CA, USA). The sections were then treated with streptavidin peroxidase (TS125HR, Thermo Scientific Lab Vision, Fremont, CA, USA) and a brown color developed with 3,3′-diaminobenzidine substrate (TA125QHDX, Thermo Scientific Lab Vision). The sections were then counterstained with hematoxylin (NM-HEM, Biocare Medical, LLC). As a negative control, adjacent serial sections were incubated with ChromPure mouse IgG and ChromPure rabbit IgG (015-000-003 and 011-000-003, Jackson Immunoresearch Labs, West Grove, PA, USA) at the same concentration as the primary antibodies. Staining for Nodal and ABCA1 were analyzed and scored blinded with respect to clinical information. Dual staining of the tissue sections with anti-Nodal and anti-ABCA1 antibodies was performed using the MACH 2 Double Stain 2 reagent according to the manufacturer’s protocols (Biocare Medical, MRCT525) with the Warp Red Chromogen kit (WR806) for red color.

### 3.3. Statistical Analyses and Clinical Correlations

Descriptive statistics were used to summarize data, including frequency distribution and percentage for categorical variables and mean with standard deviation for continuous variables. Bar plots and waterfall plots were used to demonstrate the data before and after treatment (Figure 3A,B). In the correlative analysis between treatment and clinical outcomes, Wilcoxon signed-rank test was used to assess the change of Nodal staining and tumor size for the paired data before and after treatment.

## 4. Conclusions

In aggressive breast cancer, where a lack of targetable molecules exists, together with the likelihood for relapse following chemotherapy, additional studies are needed to evaluate novel biomarkers associated with disease progression and drug resistance. Our discovery of the reactivation of the Nodal signaling pathway in cancer has provided new insights—and instigated additional questions—into the linkage that appears to exist among the CSC phenotype, disease progression, and drug resistance, which can inform the design of more effective clinical trials. Indeed, targeting CSCs and their metastatic niches presents new therapeutic opportunities worth pursuing based on an accumulating body of evidence [28,29].

We recognize that normal progenitor cells and CSCs use similar signaling pathways to sustain growth. Moreover, findings related to the convergence of embryonic and tumorigenic signaling pathways have illuminated the significance of oncofetal targets that are strictly regulated during normal development but aberrantly reactivated in aggressive forms of cancer [18,30]. Particularly noteworthy are oncofetal targets, such as Nodal, that re-emerge only in aggressive cancers but not in normal tissues. The work presented in this paper confirms previous studies showing the importance of Nodal as a CSC molecule associated with aggressive breast cancer, and advances the field by providing new findings indicating that Nodal is not targeted by standard-of-care therapy in breast cancer patients. Most noteworthy is the linkage between Nodal expression and the drug resistance marker ABCA1. Although the results are based on a small sample number, the preliminary findings are of special interest in the design of new therapeutic strategies that target the stem cell properties of adult cancer cells, especially as part of a combinatorial approach to overcome drug resistance and disease recurrence.

## Figures and Tables

**Table 1:** Specific diagnoses, clinical measurements, treatment regimens, and observations for the breast cancer patients’ tumors, with incomplete pathological responses, examined in this study.

Subject Number	Age at Diagnosis (years)	Histology	Grade	ER%	PR%	Her2/neu	Ki67	Clinical Stage	Original Tumor Size	* Lymph Node Involvement Pre-Therapy	Pathologic Stage
**SH-02**	53	Ductal	3	0	0	1	56	IIA	2.8	N0	I
**SH-04**	69	Ductal	2	99	22	1	33	IIIB	4.9	N1	IIA
**SH-05**	67	Ductal	2	100	100	1	13	IIIB	5.1	N1	IIIB
**SH-08**	32	Ductal	3	0	0	1	86	IIB	5.1	N0	I
**SH-09**	56	Ductal	2	100	62	2 negatives FISH	24	IIB	2.7	N1	IIA
**SH-10**	45	Ductal	2	99	96	1	31	IIA	1.8	N1	IIA
**SH-11**	58	Ductal	3	0	0	3	97	IIB	2.4	N1	IIB
**SH-12**	45	Ductal	3	95	88	2 positives FISH	74	IIA	3.3	N1	I
**SH-13**	53	Ductal	3	0	0	1	90	IIIA	2.6	N2	IIA
**SH-14**	57	Ductal	2	100	100	1	43	IIIC	2.8	N3	IIB
**SH-15**	59	Ductal	2	100	34	1	25	III	3.5	N1	IIIC
**SH-17**	61	Ductal	3	5	0	1	89	IIB	3.4	N1	I
**SH-19**	59	Ductal	3	22	0	1	96	IIB	3.7	N1	IIA
**SH-20**	42	Ductal	3	5	3	1	ND	IIB	2.6	N1	IIIC

* Lymph node status categories. N0: Axillary and other nearby lymph nodes do not have cancer or only have isolated tumor cells (individual cancer cells), when examined under a microscope; N1: Micrometastases (very small clusters of cancer cells), or 1–3 axillary lymph nodes have cancer, and/or internal mammary nodes have cancer or micrometastases found on sentinel node biopsy; N2: 4–9 axillary lymph nodes have cancer, or internal mammary nodes have cancer, but axillary lymph nodes do not have cancer; N3: 10 or more axillary lymph nodes have cancer, or infraclavicular (under the clavicle) nodes have cancer, or internal mammary nodes have cancer plus 1, or more axillary lymph nodes have cancer, or 4 or more axillary lymph nodes have cancer plus internal mammary nodes have cancer, or micrometastases found on sentinel node biopsy, or supraclavicular (above the clavicle) nodes have cancer. ER: estrogen receptor; PR: progesterone.

**Table 2:** Specific diagnoses, clinical measurements, treatment regimens and observations for the breast cancer patients’ tumors, with incomplete pathological responses, examined in this study.

Subject Number	^†^ %Nodal Expression in Tumor Pre-Treatment	^†^ %Nodal Expression in Tumor Post-Treatment	^†^ %ABCA1 Expression in Tumor Pre-Treatment	^†^ %ABCA1 Expression in Tumor Post-Treatment	Post-Therapy Tumor Size	* Lymph Node Involvement after Therapy	Neo-Adjuvant Therapy (Drug Regiment)	Surgical Intervention	Distant Recurrence	Years Since Diagnosis
**SH-02**	25%	95%	30%	80%	0.9	N0	ACx4;Tx12	Lump and SLN	No	2
**SH-04**	95%	90%	50%	80%	2.4	N0	ACx4;Tx10	Mastectomy and ALND	No	1
**SH-05**	50%	95%	25%	80%	5	N1	TC x 4	Mastectomy and ALND	No	2
**SH-08**	90%	40%	0%	75%	0.6	N0	ACx4;Tx12	Mastectomy and SLN	No	2
**SH-09**	95%	75%	95%	95%	0.5	N1	ACx4;Tx12	Mastectomy and ALND	No	3
**SH-10**	90%	95%	90%	80%	1.5	N1	ACx4;Tx12	Central lumpectomy and SLN	No	1
**SH-11**	90%	95%	10%	60%	1.9	N1	TCHP	Lump and SLN	No	1
**SH-12**	95%	90%	10%	80%	0.7	N0	TCHP	Lump and SLN	No	1
**SH-13**	10%	95%	75%	40%	2.2	N1	ACx4;Tx12	Lump and ALND	No	1
**SH-14**	75%	90%	20%	20%	2.8	N1	TCx4	Mastectomy and ALND	No	2
**SH-15**	95%	95%	20%	95%	2.2	N3	ACx4;Tx12	Mastectomy and ALND	No	1
**SH-17**	80%	75%	80%	80%	1.4	N0	ACx4;Tx12	Lump and SLN	Yes	2
**SH-19**	50%	90%	10%	90%	2.6	N0	ACx4;Tx2	Lump and ALND	No	1
**SH-20**	75%	80%	10%	50%	6.3	N3	TCx6	Mastectomy and ALND	Yes	2

* Lymph node status categories. N0: Axillary and other nearby lymph nodes do not have cancer or only have isolated tumor cells (individual cancer cells), when examined under a microscope; N1: Micrometastases (very small clusters of cancer, or 1–3 axillary lymph nodes have cancer, and/or internal mammary nodes have cancer or micrometastases found on sentinel node biopsy; N2: 4–9 axillary lymph nodes have cancer, or internal mammary nodes have cancer, but axillary lymph nodes do not have cancer; N3: 10 or more axillary lymph nodes have cancer, or infraclavicular (under the clavicle) nodes have cancer, or internal mammary nodes have cancer plus 1, or more axillary lymph nodes have cancer, or 4 or more axillary lymph nodes have cancer plus internal mammary nodes have cancer, or micrometastases found on sentinel node biopsy, or supraclavicular (above the clavicle) nodes have cancer; † %Nodal (or † %ATP-binding cassette member 1 (ABCA1)) in the tumor is relative to Nodal (or ABCA1) measured in the entire tumor, where 100% represents every tumor cell expressing Nodal (or ABCA1). ACT: (doxorubicin/cyclophosphamide/taxanes; TCHP: docetaxel/carboplatin/trastuzumab/pertuzumab; TC: docetaxel/cyclophosphamide; SLN: sentinel lymph node; ALND: ancillary lymph node dissection.

## Supplementary Materials

The following are available online at https://www.mdpi.com/2072-6694/11/3/340/s1, Figure S1: Breast cancer patient tumor sections, pre- and post-current standard-of-care-treatments (ACT, TCHP and TC), were examined by dual-immunohistochemical staining for Nodal (brown stain) and ABCA1 (red stain).
