# Correction: Margaryan et al. The Stem Cell Phenotype of Aggressive Breast Cancer Cells. *Cancers* 2019, *11*, 340

**DOI:** 10.3390/cancers17223701

**Published:** 2025-11-19

**Authors:** Naira V. Margaryan, Hannah Hazard-Jenkins, Mohamad A. Salkeni, Matthew B. Smolkin, James A. Coad, Sijin Wen, Elisabeth A. Seftor, Richard E. B. Seftor, Mary J. C. Hendrix

**Affiliations:** 1 Department of Biochemistry, West Virginia University, Morgantown, WV 26506, USA; naira.margaryan@hsc.wvu.edu (N.V.M.); elisabeth.seftor@hsc.wvu.edu (E.A.S.); richard.seftor@hsc.wvu.edu (R.E.B.S.); 2 West Virginia University Cancer Institute, West Virginia University, Morgantown, WV 26506, USA; hhazard@hsc.wvu.edu (H.H.-J.); mosalkeni@hsc.wvu.edu (M.A.S.); mbsmolkin@hsc.wvu.edu (M.B.S.); jcoad@hsc.wvu.edu (J.A.C.); siwen@hsc.wvu.edu (S.W.); 3 Department of Surgery, West Virginia University, Morgantown, WV 26506, USA; 4 Department of Internal Medicine, West Virginia University, Morgantown, WV 26506, USA; 5 Department of Pathology, Anatomy and Laboratory Medicine, West Virginia University School of Medicine, Morgantown, WV 26506, USA; 6 Department of Biostatistics, School of Public Health, West Virginia University, Morgantown, WV 26506, USA; 7 Department of Biology, Shepherd University, Shepherdstown, WV 25443, USA

## Error in Figure

In the original publication [1], there was a mistake in Figure 4 as published. Figure 4 was found to have incorrectly duplicated the post-treatment IgG (control) image in place of the SH-11 pre-TCHP image. All original images for this paper were reviewed and the correct SH-11 pre-TCHP image representing the SH-11 pre-TCHP sample has been inserted in Figure 4. The corrected Figure 4 appears below. The authors confirm that the scientific conclusions are unaffected. This correction was approved by the Academic Editor. The original publication has also been updated.

## Figures and Tables

**Figure 4:**
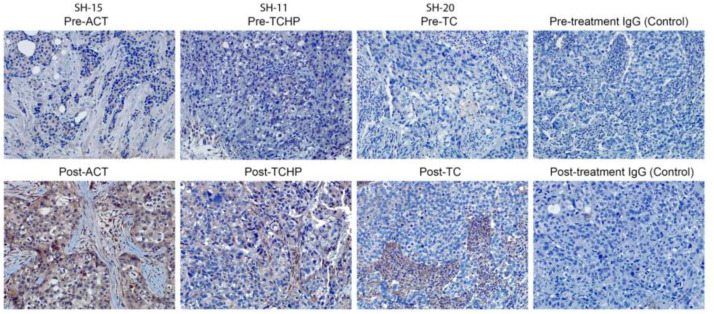
The presence of the ABCA1 protein (brown color) in breast cancer patient tumor sections, pre- and post-current-standard-of-care-treatments (ACT, TCHP, and TC) was examined by immunohistochemical staining with IgG used as a control for non-specific staining (20× original magnification).
